# Association of Atrial Fibrillation With Patient Characteristics in Postoperative Coronary Artery Bypass Grafting Surgery

**DOI:** 10.7759/cureus.19800

**Published:** 2021-11-22

**Authors:** Hafiz Ali S Rajput, Faryal Khan, Uzair Qayum Zargar, Fizza Iqbal, Khizer Shamim, Abdul Wahab, Izza Khalid, Zainab Siddiqui, Laraib S Rajput, Kiran Abbas

**Affiliations:** 1 Department of Medicine, Liaquat National Hospital, Karachi, PAK; 2 Department of Internal Medicine, Jinnah Sindh Medical University, Karachi, PAK; 3 Department of Medicine, Jinnah Postgraduate Medical Centre, Karachi, PAK; 4 Department of Medicine, Dow Medical College, Karachi, PAK; 5 Department of Medicine, Ziauddin University, Karachi, PAK; 6 Department of Medicine, Abbasi Shaheed Hospital, Karachi, PAK; 7 Department of Medicine, Fatima Jinnah Medical University, Lahore, PAK; 8 Department of Medicine, Dow University Hospital, Karachi, PAK

**Keywords:** arrhythmias, intensive care unit, coronary artery bypass grafting surgery, coronary artery bypass surgery, atrial fibrillation

## Abstract

Introduction

Atrial fibrillation (AF) after coronary artery bypass grafting (CABG) is correlated with poor patient outcomes. The study evaluated the association of patients' clinical and sociodemographic characteristics with the incidence of atrial fibrillation, postoperatively.

Methodology

A longitudinal study was performed in the cardiology department of a tertiary care unit, Sindh, Pakistan between October 2019 and November 2020. All patients who underwent CABG surgery irrespective of gender aged 30 to 75 years were included in the study. Patients with a history of atrial fibrillation or severe left ventricular dysfunction were excluded from the study. The incidence of atrial fibrillation was determined by observing an irregular pattern on electrocardiography (ECG) with no definite P-wave and irregular R-R interval. The patients were monitored for seven postoperative days. The final outcome was measured on the seventh postoperative day.

Results

A total of 247 patients with a mean age of 63.43 ± 9.72 were enrolled in the study. Out of the 247 patients, 9.7% developed new-onset atrial fibrillation, postoperatively. Age above 65 years was associated with the occurrence of AF but it was not statistically significant (p>0.05). Similarly, patients who developed AF were more likely to have a left ventricle ejection fraction (LVEF) of less than 35% than those without AF (66.67% vs 43.95%; p=0.033).

Conclusion

A high rate of AF was observed in the study. Older age and impaired ventricular function were significantly associated with atrial fibrillation. It is recommended that hospitals should devise guidelines and protocols for the prevention and management of atrial fibrillation in patients undergoing cardiothoracic surgeries in order to minimize patient mortality and improve patient outcomes.

## Introduction

Atrial fibrillation (AF) following cardiothoracic procedures such as coronary artery bypass grafting (CABG) surgery is a serious concern for intensivists [1]. New-onset atrial fibrillation after coronary artery bypass grafting has been linked with a high risk of patient mortality and morbidity [2]. The incidence of atrial fibrillation in postoperative patients is estimated to be between 25 to 60% of patients which is ultimately dependent on several factors such as the surgical procedure and baseline characteristics of the patient [3,4]. Amar et al. revealed increasing age as the most significant factor in evaluating the risk for atrial fibrillation among patients who underwent elective thoracic surgery. The authors identified that patients aged 60 or above (p<0.0001) and a preoperative heart rate of ≥ 74 beats/min (p<0.0007) were independent risk factors for AF [5]. Literature shows that patients who are hemodynamically unstable or symptomatic post cardiac procedures benefit from rhythm strategies while the rest are seen to benefit from rate strategies [6,7]. Prophylaxis in some studies was seen to reduce the frequency of postoperative atrial fibrillation [8]. The frequency of atrial fibrillation postoperatively is also affected by the way CABG is performed on patients. The prevalence however of AF is seen to increase postoperatively when valve replacement is performed alongside CABG [9]. The incidence of atrial fibrillation in postoperative CABG patients is not known in our population and the factors associated with it are still debatable. Therefore, the current study aimed to determine the association of patients' clinical and sociodemographic characteristics with the incidence of atrial fibrillation, postoperatively. 

## Materials and methods

A longitudinal study was performed in the cardiology department of a tertiary care unit, Sindh, Pakistan after approval from the local ethical committee (reference #JSMU/IRB/2019/-154). The study was conducted from October 2019 to November 2020. Using non-probability convenience sampling technique, the participants were enrolled. All patients who underwent CABG surgery during the study period irrespective of gender aged between 35 to 75 years were included in the study. Patients with a history of atrial fibrillation or severe left ventricular dysfunction were excluded from the study. 

Informed verbal and written consent was acquired from all the patients prior to inclusion in the study. Sociodemographic information including age, ethnicity, gender, residence, occupation, education, was documented on a pre-formed pro forma. The incidence of atrial fibrillation was determined by observing an irregularly irregular pattern on electrocardiography (ECG) with no definite P-wave and irregular R-R interval. The patients were monitored for seven postoperative days. The final outcome was measured on the seventh postoperative day. All the data were analyzed using the Statistical Package for Social Sciences (SPSS) version 23 (IBM Corp., Armonk, NY, USA). Mean and standard deviation was determined for continuous variables while categorical variables i.e. atrial fibrillation, frequency, and percentages were determined. A Chi-square test was used to find out the association between atrial fibrillation and patient characteristics. 

## Results

A total of 247 patients with a mean age of 63.43 ± 9.72 years were enrolled in the study. The majority of the patients were above 51 years. There was a male predominance in our study (Table 1). 

**Table 1:** Patient Characteristics

Characteristics	N (%)
Mean age	63.43 ± 9.72
Age (Years)	
30-50	25 (10.12%)
51-65	93 (37.65%)
65 above	129 (52.2%)
Gender	
Female	83 (33.6%)
Male	164 (66.4%)
Atrial fibrillation	
Yes	24 (9.71%)
No	223 (90.28%)

Out of the 247 patients, 9.7% developed new-onset atrial fibrillation, postoperatively. Age above 65 years was significantly associated with the occurrence of AF (P=0.05). Similarly, patients who developed AF were more likely to have a left ventricle ejection fraction (LVEF) of less than 35% than those without AF (66.67% vs 43.95%; p=0.033) (Table 2). 

**Table 2:** Association of Patient Characteristics With Occurrence of Atrial Fibrillation in Patients

Characteristics	Atrial Fibrillation		P-value
Variables	Yes (n=24)	No (n=223)	
Age			
<65	7 (29.17%)	111 (49.55%)	0.057
>65	17 (70.83%)	112 (50.45%)	
Valvular Heart Disease			
Yes	9 (37.50%)	20 (8.93%)	0.456
No	15 (62.50%)	104 (46.43%)	
Chronic Obstructive Pulmonary Disease			
Yes	10 (41.67%)	100 (44.64%)	0.78
No	14 (58.33%)	124 (55.36%)	
Electrolyte imbalance ( < 3.5 or > 5 mM/L)			
Yes	13 (54.17%)	105 (47.09%)	0.497
No	11 (45.83%)	119 (53.36%)	
Left ventricle ejection fraction (LVEF)			
> 35%	8 (33.33%)	125 (56.05%)	0.033
< 35%	16 (66.67%)	98 (43.95%)	
Left Atrial size			
> 40 mm	14 (58.33%)	103 (46.19%)	0.257
< 40 mm	10 (41.67%)	120 (53.81%)	

## Discussion

AF is a common presentation among postoperative CABG patients. The present study found that almost 10% of patients suffered from atrial fibrillation, postoperatively. Some studies have explored atrial fibrillation among patients who underwent CABG. For instance, a study by Ozcan found that 19.41% developed AF requiring pharmacological intervention. Older age and male gender were significantly associated with AF [10]. Iftikhar et al. revealed an overall occurrence of AF in postoperative cardiac patients as 6.9% [11]. The rate of AF in patients who underwent CABG in local studies was between 6% to 15%. The slight difference in the incidence of AF could be because of the varying inclusion or exclusion criteria and also the difference in age of the patients. 

Similarly, a meta-analysis was conducted by Higgs et al. in which postoperative incidence of atrial fibrillation was found to be 25% [12]. Furthermore, risk factors that led to atrial fibrillation after cardiac procedures included male gender, congestive heart failure, chronic obstructive pulmonary disease (COPD), reduced partial pressure of oxygen, and right coronary artery disease. Matos et al. however in their study state that anticoagulation after CABG in new-onset AF has been seen with increased risk of bleeding but no significant variation in risk of stroke on day 30 [13]. Previous studies have also highlighted that CABG use or the type of cardiac surgery being performed determines the risk of atrial fibrillation postoperatively [14,15]. Furthermore, atrial fibrillation occurring after a cardiac procedure was linked to one-year mortality rate and longer length of stay in the hospital [15]. Khair et al. in their study found that the age of patients having postoperative atrial fibrillation after the CABG procedure was higher (62 years) than those who did not have atrial fibrillation (54 years, p< 0.05) [16]. Another study by Banach et al. concluded that the most important factor leading to increased risk of postoperative atrial fibrillation in patients was age [17]. Furthermore, the risk of postoperative atrial fibrillation was not seen to be higher in patients with comorbidities such as hypertension, diabetes mellitus, renal failure, and heart failure than those without atrial fibrillation [18]. Our study was not without limitations. Due to the limited sample size and monocentric nature of the study, the findings of the study could not be generalized to a larger population. Further large-scale studies with varying demographics and detailed clinical history can increase the weight of future studies.

## Conclusions

The present study detected atrial fibrillation in approximately 10% of patients who underwent CABG. Atrial fibrillation is an independent factor for in-hospital mortality. Older age and impaired ventricular function were significantly associated with atrial fibrillation. It is recommended that hospitals should devise guidelines and protocols for the prevention and management of atrial fibrillation in patients undergoing cardiothoracic surgeries in order to minimize patient mortality and improve patient outcomes.
